# Decrease health-related quality of life in pediatric leprosy patients with musculoskeletal manifestations

**DOI:** 10.1186/1546-0096-12-S1-P38

**Published:** 2014-09-17

**Authors:** Luciana Neder, Marlon van Weelden, Gabriela R Viola, Daniela M Lourenço, Claudio A Len, Clovis AA Silva

**Affiliations:** 1 Pediatric Rheumatology Unit, Faculdade de Medicina da Universidade de São Paulo, Brazil; 2 Pediatric Rheumatology Unit, Unifesp, São Paulo, Brazil

## Introduction

Leprosy, also known as Hansen’s disease, is caused by the *Mycobacterium leprae*. The clinical features of pediatric leprosy include several skin lesions, numbness of the skin, damage of peripheral nerves, arthralgia and arthritis. In this regard, we recently reported that musculoskeletal manifestations were associated with severe leprosy in children and adolescents, especially in patients presenting nerve function impairment and neuropathy. Furthermore, adult leprosy patients could present a decrease in health-related quality of life (HRQL), particularly in physical capacity and social participation domains. To our knowledge, HRQL was rarely reported in pediatric leprosy, and the impact of musculoskeletal manifestations on HRQL was not previous investigated.

## Objectives

To evaluate the HRQL in pediatric leprosy patients.

## Methods

A cross-sectional study included 47 leprosy patients and 45 healthy subjects. The HRQL was measured by Pediatric Quality of Life Inventory 4.0 (PedsQL 4.0), and evaluated physical, emotional, social and school domains. The leprosy patients were classified by Ridley and Jopling classification criteria and assessed according to clinical musculoskeletal manifestations, laboratory and radiographic examinations.

## Results

The median of current age was similar in leprosy patients and controls [12 (6-18) vs. 15 (5-18) years, p=0.384], likewise the frequencies of female gender (p=0.835) and middle/lower Brazilian socio-economic classes (p=1.0). The domain school activities according the child-self report was significantly lower in leprosy patients compared to controls in the age group of 13-18 years [75 (45-100) vs. 90 (45-100), p=0.021]. The other domains were alike in both groups (p>0.05). At least one musculoskeletal manifestation (arthralgia, arthritis and/or myalgia) was observed in 15% of leprosy patients and none in controls (p=0.012). Further comparison between all leprosy patients showed that the median of the physical capacity domain [81.25 (50-100) vs. 98.44 (50-100), p=0.036] and school activities domain by child-self report [60 (50-85) vs. 80 (45-100), p=0.042] were significantly lower in patients with musculoskeletal manifestations compared to patients without these manifestations. No differences were evidenced between the other HRQL parameters in both groups, reported by patients and parents (p>0.05).

## Conclusion

A reduced HRQL was observed in pediatric leprosy patients with musculoskeletal manifestations. Specific interventions in physical and school activities are required to improve HRQL in this high-risk population.

## Disclosure of interest

L. Neder: None declared., M. van Weelden: None declared., G. R. Viola: None declared., D. M. Lourenço: None declared., C. A. Len: None declared., C. A. Silva Grant / Research Support from: This study was supported by Fundação de Amparo à Pesquisa do Estado de São Paulo (FAPESP - grants 2008/58238-4 to CAS), by Conselho Nacional do Desenvolvimento Científico e Tecnológico (CNPQ – grant 302724/2011-7 to CAS), by Federico Foundation to CAS and by Núcleo de Apoio à Pesquisa “Saúde da Criança e do Adolescente” da USP (NAP-CriAd).

